# Roflumilast Enhances Liraglutide’s Atrial Natriuretic Peptide-Dependent Suppression of Adrenal Aldosterone Secretion

**DOI:** 10.3390/ijms27094098

**Published:** 2026-05-03

**Authors:** Ariana Hosseini, Alexis J. M’Sadoques, Renee A. Stoicovy, Victoria L. Altsman, Laura Raynshteyn, Emma Weinstein, Teresa Baggio Lopez, Giselle Del Calvo, Madyson G. Leiker, Anastasios Lymperopoulos

**Affiliations:** Laboratory for the Study of Neurohormonal Control of the Circulation, Department of Pharmaceutical Sciences (Pharmacology), Barry and Judy Silverman College of Pharmacy, Nova Southeastern University, Fort Lauderdale, FL 33328-2018, USA; ah3322@mynsu.nova.edu (A.H.); am4441@mynsu.nova.edu (A.J.M.); rs2981@mynsu.nova.edu (R.A.S.); va566@mynsu.nova.edu (V.L.A.); lr1940@mynsu.nova.edu (L.R.); ew921@mynsu.nova.edu (E.W.); tb1985@mynsu.nova.edu (T.B.L.); gd849@mynsu.nova.edu (G.D.C.); ml3196@mynsu.nova.edu (M.G.L.)

**Keywords:** adrenocortical cells, aldosterone, angiotensin II, atrial natriuretic peptide, cardiac myocyte, GLP-1 receptor agonist, liraglutide, phosphodiesterase-4 inhibitor, signal transduction

## Abstract

Glucagon-like peptide (GLP)-1 receptor (GLP1R) agonists exert a multitude of beneficial cardiovascular effects beyond control of blood glucose levels and obesity reduction. GLP-1R is a G protein-coupled receptor (GPCR), coupling to adenylyl cyclase (AC)-stimulatory Gs proteins to raise cyclic 3′-5′-adenosine monophosphate (cAMP) levels in cells. cAMP exerts various effects mainly via protein kinase A (PKA) and Exchange protein directly activated by cAMP (Epac). Cardiac GLP-1R has been reported to induce atrial natriuretic peptide (ANP) secretion via Epac2, while ANP is known to inhibit aldosterone secretion from adrenocortical zona glomerulosa (AZG) cells. Herein, we tested the effects of the GLP-1R agonist liraglutide on ANP secretion in H9c2 cardiomyocytes and on angiotensin II (AngII)-induced aldosterone secretion. We also examined whether phosphodiesterase (PDE)-4 inhibition with roflumilast could potentiate liraglutide’s effects. We found that liraglutide stimulated ANP secretion from H9c2 cardiomyocytes, an effect potentiated by roflumilast but blocked by AC inhibition. Epac inhibition with ESI-09 also significantly reduced liraglutide-dependent ANP secretion in H9c2 cardiomyocytes. Moreover, application of medium from liraglutide-treated H9c2 cardiomyocytes, but not from control cardiomyocytes, led to suppression of AngII-dependent aldosterone secretion from H295R cells. This effect was blocked by cyclic guanosine monophosphate (cGMP)-dependent protein kinase inhibition (an effector of ANP) in H295R cells, while direct application of liraglutide to these cells failed to suppress AngII-induced aldosterone secretion. Again, aldosterone suppression was more potent when medium from liraglutide plus roflumilast-treated cardiomyocytes was applied to H295R cells. Taken together, these results suggest that roflumilast enhances the adrenocortical aldosterone suppression induced by GLP-1R agonists via cardiac GLP-1R/cAMP/Epac-dependent ANP secretion. Given the cardio-toxic effects of elevated aldosterone levels in the context of various heart diseases, such as post-myocardial infarction heart failure, combination of a GLP-1R agonist drug with a PDE4 inhibitor drug may be more advantageous than either agent alone in treatment of certain cardiovascular diseases.

## 1. Introduction

Agonists of the glucagon-like peptide-1 (GLP-1) receptor (GLP-1R) are important drugs used in diabetes and obesity [1,2,3]. They also demonstrate a variety of direct cardiovascular benefits, many of which derive from systemic and local, tissue-specific anti-inflammatory actions, including cardiomyocytes, and are independent of their anti-diabetic or weight loss effects [3,4,5,6,7,8,9]. GLP-1R is a class B G protein-coupled receptor (GPCR) (specifically, a B1/secretin GPCR subfamily member), coupling to the stimulatory Gs type of heterotrimeric G proteins [10]. This results in adenylyl cyclase (AC)-mediated production of the second messenger cyclic 3′, 5′-adenosine monophosphate (cAMP) [10], which exerts its effects inside cells mainly via protein kinase A (PKA) and Exchange protein directly activated by cAMP (Epac)-1/2 [11,12]. GLP-1R is expressed in the myocardium, in particular in atrial myocardium, wherein it exerts positive chronotropy (increases heart rate) via stimulation of cAMP synthesis in sinoatrial nodal cells [13,14].

GLP-1R has also been reported to stimulate atrial natriuretic peptide (ANP) secretion from atrial myocytes via Epac2 [15] and from lungs of various species [16,17], although a definitive effect of GLP-1R agonists on ANP secretion has yet to be confirmed in humans [18]. ANP is a major heart-secreted hormone that promotes, among other beneficial effects for the cardiovascular system, lowering of blood pressure and natriuresis [19], two effects shared by GLP-1R agonist drugs like liraglutide, as well [20,21,22]. One of the mechanisms by which ANP lowers blood pressure and induces sodium excretion is direct inhibition of angiotensin II (AngII)-dependent adrenal aldosterone production in adrenocortical zona glomerulosa (AZG) cells [23]. GLP-1R agonists are also known to lower aldosterone levels, at least acutely [24,25,26,27,28,29,30], since due to their hypotensive and natriuretic effects, they may actually increase aldosterone levels long term [31]. However, whether the mechanisms of GLP-1R agonist-induced aldosterone suppression are direct (at the level of adrenocortical aldosterone production) or indirect (secondary to renin and AngII suppression) or both is still under investigation. Since cardiac GLP-1R-induced ANP secretion could be another mechanism for GLP-1R agonists’ hypo-aldosteronic effect, we investigated in the present study whether liraglutide treatment of cardiac cells and subsequent application of the supernatant medium to human AZG cells results in reduced aldosterone secretion in the latter. We also recently reported that the cAMP-selective phosphodiesterase type 4 (PDE4) inhibitor roflumilast enhances liraglutide’s protective effects against inflammatory injury in H9c2 cardiomyocytes [8]. Thus, we also examined whether roflumilast can potentiate the effect of liraglutide on aldosterone secretion from AZG cells, as well.

## 2. Results

### 2.1. Liraglutide Induces Anp Secretion via Camp and Partly via Epac in H9c2 Cardiomyocytes

**Figure 1:**
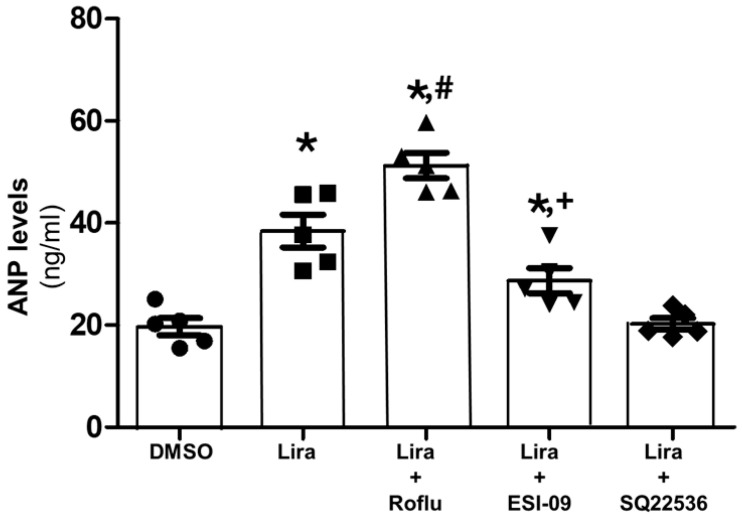
Liraglutide-induced ANP secretion in H9c2 cardiomyocytes. ANP levels in the supernatant medium of H9c2 cardiomyocytes at 30 min post-treatment with vehicle (0.5% DMSO) or 200 nM liraglutide (Lira) with or without 5 µM roflumilast (Roflu), 1 µM ESI-09 (Epac1/2 inhibitor), or 200 µM SQ22536 (AC inhibitor). Data are expressed as fold of vehicle. *, *p* < 0.05, vs. vehicle; ^#^, *p* < 0.05, vs. Lira; ^+^, *p* < 0.05, vs. Lira; n = 5 independent experiments per treatment condition.

Despite not being bona fide contracting cardiomyocytes, H9c2 cells can produce and secrete ANP [32,33,34]. Thus, we first examined whether the endogenous GLP-1R [8,35,36,37] has any effects on ANP secretion in H9c2 cardiomyocytes. As shown in Figure 1, acute (30 min) treatment of liraglutide resulted in ANP secretion into the cell culture medium. Importantly, this effect was enhanced in the presence of roflumilast and completely abrogated by AC pharmacological inhibition with SQ22536 (Figure 1). Additionally, addition of the Epac1/2 pharmacological inhibitor ESI-09 significantly reduced, but not completely blocked, liraglutide-induced ANP secretion (Figure 1). These results strongly suggest that cardiac GLP-1R can induce ANP secretion via stimulation of cAMP synthesis, which, in turn, stimulates ANP secretory granule membrane fusion/exocytosis in part via Epac, as previously reported [15], but also probably via PKA.

### 2.2. Liraglutide Has No Direct Effect in h925r AZG Cells

**Figure 2:**
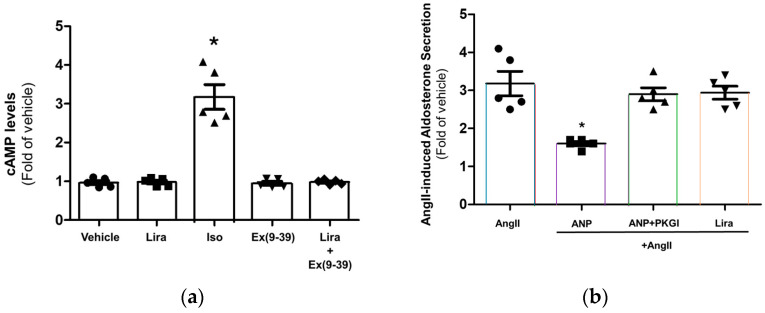
No direct effect of liraglutide in AZG cells. (**a**) cAMP levels in H295R cells in response to vehicle (0.5% DMSO), 200 nM liraglutide (Lira), 1 µM isoproterenol (Iso), 200 nM exendin(9-39) [Ex(9-39)] (GLP-1R antagonist), or 200 nM liraglutide + 200 nM exendin(9-39) [Lira+ Ex(9-39)] for 30 min; (**b**) Aldosterone secretion from H295R cells in response to a 100 nM AngII challenge for 1 h alone or in the presence of 1 µM ANP (ANP+AngII), or of 1 µM ANP with 10 µM PKG inhibitor (ANP+PKGI+AngII), or of 200 nM liraglutide (Lira+AngII). In both panels, data are expressed as fold of vehicle (0.5% DMSO). *, *p* < 0.05, vs. vehicle; n = 5 independent experiments per treatment condition.

Next, we examined the effects of GLP-1R agonism with liraglutide in the human AZG cell line H295R, which expresses the AngII type 1 receptor (AT_1_R) endogenously and secretes aldosterone in response to AngII [38]. Liraglutide failed to stimulate cAMP synthesis in H295R cells (Figure 2a), and application of the GLP-1R inhibitor exendin(9-39) alone or together with liraglutide had no effect, either (Figure 2a). In contrast, the β-adrenergic receptor (AR) agonist isoproterenol produced a robust increase in cAMP levels, consistent with the previously reported presence of endogenous βARs in H295R cells [39].

In addition, liraglutide had no effect on aldosterone secretion from H295R cells, either when applied alone or on AngII-induced aldosterone secretion (Aldosterone secretion with AngII alone: 401 ± 40.1 pg/mL, vs. with liraglutide+AngII: 363 ± 23.1 pg/mL) (Figure 2b). In contrast, ANP significantly (but incompletely) suppressed AngII-induced aldosterone secretion (Figure 2b), as previously reported [23] (Aldosterone secretion with AngII+ANP: 200.5 ± 7.2 pg/mL). Of note, the ANP-mediated aldosterone suppression was completely dependent on cyclic guanosine monophosphate (cGMP) signaling and activation of protein kinase G (PKG) by the ANP receptor (NPR-A or membrane-bound guanylyl cyclase A, GC-A), endogenously expressed in H295R cells [40], since pharmacological PKG inhibition (with the RQIKIWFQNRRMKWKKLRKKKKKH peptide [41]) prevented ANP from inhibiting aldosterone secretion in response to AngII (Aldosterone secretion with AngII+ANP+PKG inhibitor: 364 ± 22 pg/mL, Figure 2b). Taken together, these results indicate that GLP-1R agonism has no effects in H295R cells, probably because GLP-1R is absent in this cell type. Indeed, Western blotting in H295R cells confirms that GLP-1R protein is absent from this cell line (Supplementary Figure S1).

### 2.3. Roflumilast Enhances Cardiac GLP-1R-Dependent Suppression of Aldosterone Secretion from AZG Cells via ANP

**Figure 3:**
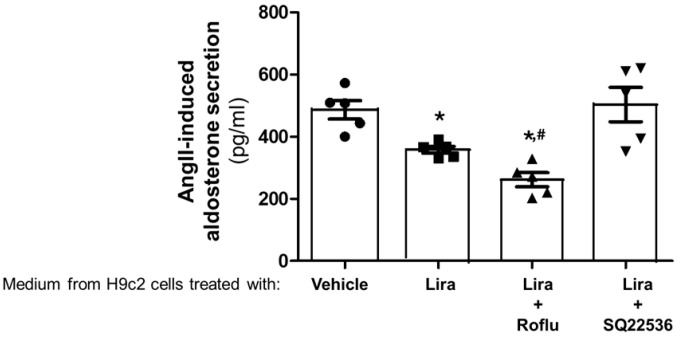
AngII-induced aldosterone secretion in AZG cells cultured in media from liraglutide-treated cardiomyocytes. Aldosterone secretion from H295R cells in response to a 100 nM AngII challenge for 1 h, placed in medium derived from vehicle (0.5% DMSO)-treated, 200 nM liraglutide (Lira)-treated, 200 nM liraglutide + 5 µM roflumilast (Lira+Roflu)-treated, or 200 nM liraglutide + 200 µM SQ22536 (Lira+SQ22536)-treated H9c2 cardiomyocytes. *, *p* < 0.05, vs. vehicle; ^#^, *p* < 0.05, vs. Lira; n = 5 independent experiments per treatment condition.

Since liraglutide does not affect aldosterone secretion from AZG cells directly, but stimulates ANP secretion from cardiomyocytes, we next tested whether ANP secreted from liraglutide-treated H9c2 cardiomyocytes can suppress aldosterone in AZG cells. To that end, we removed the media from H9c2 cardiomyocytes at the end of the liraglutide treatments and immediately applied them to H295R cells in culture prior to an AngII challenge. As expected, AngII-induced aldosterone release was partially but significantly suppressed in cells placed in liraglutide-treated cardiomyocyte medium and was further suppressed in H295R cells placed in medium from cardiomyocytes treated with liraglutide in the presence of roflumilast (Figure 3). This probably reflects the higher concentration of ANP in that medium compared to the medium from cardiomyocytes treated with liraglutide only (see Figure 1). Finally, as expected, aldosterone suppression in cells placed in medium from cardiomyocytes treated with liraglutide in the presence of AC inhibition was essentially unaffected (Figure 3), reflecting the absence of ANP in that medium (again, see Figure 1).

### 2.4. Combining ANP with Inhibition of βarrestins Results in Full Suppression of Aldosterone Secretion from AZG Cells

**Figure 4:**
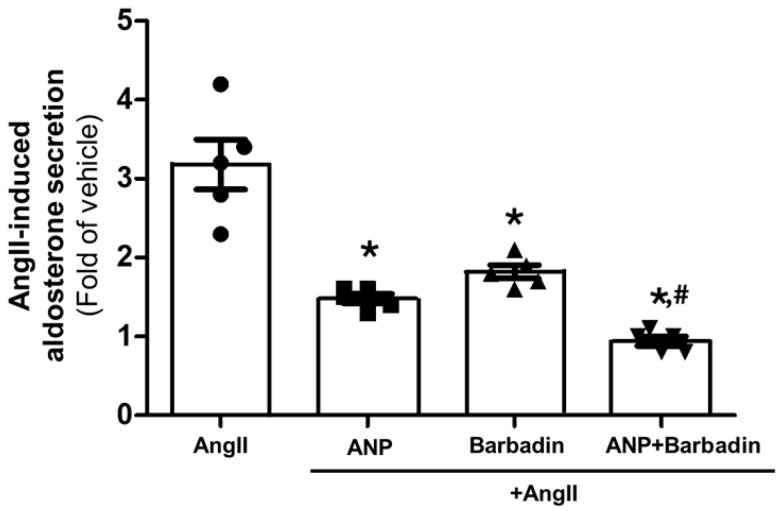
The combination of ANP with βarrestin inhibition completely abrogates AngII-induced aldosterone secretion in AZG cells. Aldosterone secretion from H295R cells in response to a 100 nM AngII challenge alone or in the presence of 1 µM ANP (ANP+AngII), or of 20 µM barbadin (Barbadin+AngII), or of 1 µM ANP with 20 µM barbadin (ANP+Barbadin+AngII). Data are expressed as fold of vehicle (no AngII challenge). *, *p* < 0.05, vs. AngII; ^#^, *p* < 0.05, vs. ANP or Barbadin; n = 5 independent experiments per treatment condition.

It is evident from the results of Figure 1 and Figure 3 above that even the highest ANP concentrations, achieved with the combination of liraglutide plus roflumilast, are insufficient to suppress AngII-induced aldosterone secretion to basal levels. Given that AT_1_R signals through both G proteins and βarrestin1 to stimulate aldosterone secretion in AZG cells [37,42,43] but ANP should probably be able to inhibit, via cGMP/PKG, only the G protein-dependent component of AT_1_R signaling, we postulated that ANP needs to be combined with βarrestin1 inhibition to perhaps afford full aldosterone suppression in AZG cells. To verify this, we took advantage of the established pharmacological βarrestin inhibitor barbadin [44], which specifically and potently blocks the AT_1_R-βarrestin interaction. As shown in Figure 4, ANP or barbadin applied alone resulted in partial AngII-induced aldosterone suppression in H295R cells, but, when applied together, they produced complete suppression of AngII-dependent aldosterone secretion. This suggests that ANP and, by extension, GLP-1R agonists can only partially inhibit adrenal aldosterone secretion because they do not affect AT_1_R signaling to aldosterone via βarrestin1.

## 3. Discussion

**Figure 5:**
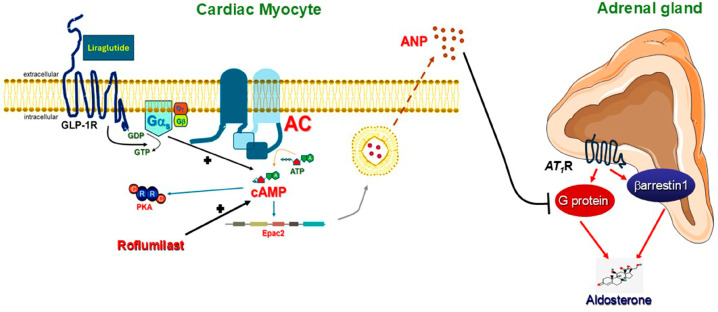
Regulation of adrenal aldosterone secretion by cardiac GLP-1R-induced ANP. Roflumilast enhances ANP secretion and aldosterone suppression via elevation of GLP-1R-dependent cAMP levels. See text for details.

In the present study, we report that activation of cardiac GLP-1R leads to adrenal aldosterone suppression via ANP secretion from cardiac myocytes (Figure 5). However, the secreted ANP only inhibits the G_q/11_ protein component of adrenocortical AT_1_R signaling towards aldosterone secretion, leaving the βarrestin1 component unaffected (Figure 5). In addition, we show that roflumilast, by inhibiting PDE4 and elevating cAMP levels in cardiomyocytes, has the potential of boosting ANP secretion, and thus, aldosterone suppression afforded by GLP-1R agonists like liraglutide (Figure 5).

Therefore, the capability of lowering adrenal aldosterone secretion is another potentially beneficial cardiovascular property of GLP-1R agonist drugs and may in part explain the well-documented natriuretic and anti-hypertensive properties of these medications [20,21,22]. Importantly, aldosterone suppression may also contribute to the cardiovascular-specific anti-inflammatory properties of GLP-1R agonists, given that aldosterone plays a major role in cardiac adverse remodeling and in cardiac and vascular inflammation during certain disease states, such as hypertension and post-myocardial infarction heart failure progression [45,46]. Finally, it should be noted that the ANP-mediated mechanism for aldosterone suppression reported here could be only one of multiple mechanisms by which GLP-1R agonists can suppress aldosterone. For example, adipokines like leptin and adiponectin, known to be regulated by the GLP-1R, also affect adrenal aldosterone secretion and circulating aldosterone levels: leptin, which is normally suppressed by GLP-1 [47], stimulates aldosterone secretion [48], whereas adiponectin, normally elevated by GLP-1R activation [49], may counteract several of the actions of aldosterone particularly in metabolic tissues under conditions of insulin resistance [50].

Another interesting finding of our present study is the apparent absence of the GLP-1R from human AZG cells (Figure 2 and Figure S1). This is consistent with the literature, as we were unable to find any studies reporting endogenous expression of this receptor in H295R cells or AZG cells of any species. Its absence from the zona glomerulosa of the adrenal cortex is also probably crucial for GLP-1R’s effect on aldosterone suppression: since GLP-1R signals through cAMP [10], and cAMP is a major signal for aldosterone secretion in AZG cells [51,52], the GLP-1R would most likely promote aldosterone secretion, rather than inhibit it, if it were expressed in AZG cells. Thus, by promoting ANP release from the heart, while being absent from the adrenocortical zona glomerulosa, GLP-1R can effectively suppress adrenal aldosterone production and lower circulating aldosterone levels. However, it is worth noting here that its apparent absence from the adrenocortical zona glomerulosa does not mean it is absent from other parts of the adrenal gland: in fact, GLP-1R has been reported to be present in the adrenal medulla, where it positively modulates catecholamine secretion from chromaffin cells [53].

The finding that liraglutide-induced ANP does not fully inhibit AngII-induced aldosterone production appears to be due to the inability of ANP and its receptor (NPR-A) to affect the βarrestin1-dependent signaling of the AT_1_R in AZG cells. This is somewhat expected, given that ANP has been reported to inhibit aldosterone secretion via PKG-mediated inhibition of calcium influx, via AC inhibition through Gi proteins, and via recruitment of Regulator of G protein Signaling (RGS)-4, which inactivates Gα_q/11_, the G protein subunit responsible for calcium-dependent exocytosis/secretion in AZG cells that AT_1_R couples to [23,54,55]. In other words, ANP can fully inhibit AT_1_R G protein signaling but has no reported effects on βarrestin signaling. In contrast, certain AT_1_R antagonists (angiotensin receptor blockers, ARBs) can fully suppress both (G protein and βarrestin) components of AT_1_R signaling towards aldosterone production [43], thereby conferring full aldosterone suppression. Indeed, the combination of ARB with ANP was found to be more effective than ANP alone at inhibiting aldosterone production in AZG cells [23]. This means that ANP, and consequently also GLP-1R agonists, may not be very effective at reducing hyperaldosteronism in disease states where adrenal βarrestin1 expression or activity is elevated, such as in tobacco-related heart disease or in chronic heart failure [56,57].

Finally, roflumilast significantly enhances liraglutide-induced ANP levels and aldosterone reduction, thanks to elevation of cAMP levels produced by the cardiac GLP-1R. We recently reported that this PDE4-selective inhibitor also augments cardiac GLP-1R’s anti-inflammatory efficacy against bacterial endotoxin-induced inflammatory injury [8]. Thus, the therapeutic goal of mitigating elevated aldosterone levels seems to be one more pharmacological action of GLP-1R agonist drugs that can get a significant boost by its combination with PDE4 inhibition. Indeed, PDE4 inhibitor drugs, currently used mainly in treatment of certain inflammatory disorders, have been increasingly emerging as potential adjunct treatments for obesity, diabetes, and other diseases, for which GLP-1R agonists are indicated [58]. Interestingly, GLP-1R agonists might be useful in disorders for which PDE4 inhibitors are indicated: for instance, liraglutide and other GLP-1R agonists have shown to be of therapeutic value in psoriasis, the primary indication of apremilast and other PDE4-selective inhibitors [59]. Given the essential role of cAMP in GLP-1R agonists’ therapeutic efficacy and that PDE4 might be upregulated in certain inflammatory states and in diabetic cardiomyopathy [58], therapeutic combinations of GLP-1R agonists with PDE4 inhibitors might not only prove useful in boosting the clinical efficacy of the GLP-1 analogs but possibly also necessary to maintain it.

The present study has several limitations. First, it was done exclusively in cultured cells in vitro, so its findings need corroboration through in vivo investigations. Another limitation is the use of the H9c2 cell line, which are cells that are neither human (it is of rat origin), nor bona fide contracting cardiomyocytes. The other cell line used, H295R, are human AZG cells but they are also immortalized, so they are not identical to normal AZG cells, either. On the flip side, the use of native (non-genetically manipulated) and physiologically relevant cell types is among the strengths of our study.

In conclusion, we report herein that GLP-1R agonists can suppress adrenal aldosterone production via ANP secreted in response to cardiac GLP-1R activation. Since GLP-1R-induced ANP secretion is cAMP-dependent, PDE4 inhibitors like roflumilast can enhance it, thereby potentiating the hypo-aldosteronic effect of GLP-1R agonists. Aldosterone suppression contributes to the anti-inflammatory and other cardiovascular benefits of the GLP-1 class of medications, which may also be independent of their effects on weight loss and glucose metabolism.

## 4. Materials and Methods

### 4.1. Materials

Unless otherwise stated, all drugs and chemicals were from Sigma-Aldrich (St. Louis, MO, USA).

### 4.2. Cell Lines and Culture

The H9c2 rat cardiomyoblast cell line was purchased from American Type Culture Collection (Manassas, VA, USA) and cultured as previously described [8,60]. Briefly, H9c2 (embryonic rat heart-derived myoblast) cells were cultured in Dulbecco’s modified Eagle medium (DMEM; GIBCO Laboratories, Grand Island, New York, NY, USA) supplemented with 10% fetal bovine serum (FBS), streptomycin (100 mg/mL) and penicillin (100 units/mL) in a humidified incubator under the condition of 37 °C, 95% air and 5% CO_2_. H295R cells were also from the American Type Culture Collection (Manassas, VA, USA; RRID: CVCL_0458) and cultured as previously described [38,39]. Cells used for all experiments were not passaged more than 3 times (passage < 3).

### 4.3. ELISA Assays for ANP and Aldosterone Measurements

In vitro ANP secretion in the culture medium of H9c2 cells and aldosterone secretion in the culture medium of H295R cells were measured by enzyme immuno-assay (EIA) with a kit from RayBiotech (ANP ELISA Kit, Cat. #:EIA-ANP; RayBiotech, Peachtree Corners, GA, USA) and an Aldosterone EIA kit (Cat. #:11-AD2HU-E01; ALPCO Diagnostics, Salem, NH, USA), respectively, as described [38,39,61].

### 4.4. cAMP Measurements

cAMP accumulation was measured with the “Direct cAMP ELISA” kit (#ADI-900-066; Enzo Life Sciences, Farmingdale, NY, USA), as described previously [8]. Briefly, the assay is an enzyme-linked immunosorbent (ELISA) competitive immunoassay that uses a solution of cAMP conjugated to alkaline phosphatase, followed by a rabbit polyclonal anti-cAMP antibody solution, to quantify the cAMP content of samples. During a room temperature incubation with the sample, the cAMP in the sample completes with the cAMP in the conjugate solution for binding to the anti-cAMP antibody. At the end of the incubation, the plate is washed, leaving only bound cAMP, which is then detected by adding a color-producing substrate (p-nitrophenyl phosphate, pNpp) that generates a yellow color when reacting with the alkaline phosphatase conjugated to the bound cAMP. The amount of signal read at 405 nm is inversely proportional to the amount of cAMP in the sample.

### 4.5. Statistical Analyses

Statistical analyses were performed with Prism (GraphPad, La Jolla, CA, USA). Comparisons relied on 1-way ANOVA for 1 variable or 2-way ANOVA for two variables. Otherwise, unpaired two- tailed *t*-tests were performed. A *p* value less than (or equal) to 0.05 was considered significant. Data are generally presented as means with single point values or as mean values with *±* standard error of the mean (SEM) bars where appropriate.

## Data Availability

All source data files are available upon reasonable request to the correspondence author.

## Supplementary Materials

The following supporting information can be downloaded at: https://www.mdpi.com/article/10.3390/ijms27094098/s1.
