# Supplementary material for: Roflumilast Enhances Liraglutide’s Atrial Natriuretic Peptide-Dependent Suppression of Adrenal Aldosterone Secretion
Source: Int J Mol Sci. 2026 May 3;27(9):4098. doi: 10.3390/ijms27094098 (PMC13164327; doi:10.3390/ijms27094098)
Supplement: Supplementary file 1 [file ijms-27-04098-s001.zip › ijms-4262824-supplementary.pdf]

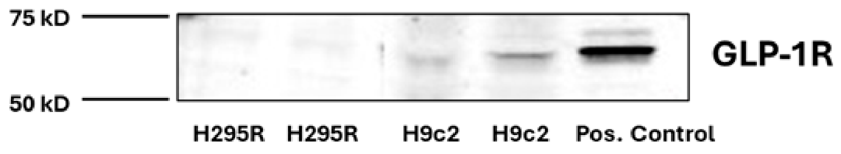

Supplementary Figure S1: Immunoblotting for GLP-1R protein expression in H295R and H9c2 cell extracts. 50 µg of protein was loaded on each lane. The last lane to the right shown is a positive control for GLP-1R protein expression (rat pancreatic beta cell extract). In contrast to H9c2 cell extracts, no GLP-1R protein could be detected in H295R cell extracts.
